# The associations between religion, bereavement and depression among Hong Kong nurses

**DOI:** 10.1186/s13104-017-2588-7

**Published:** 2017-07-04

**Authors:** Teris Cheung, Paul H. Lee, Paul S. F. Yip

**Affiliations:** 1 School of Nursing, Hong Kong Polytechnic University, Hong Kong, Hong Kong SAR, China; 2 Centre for Suicide Research and Prevention, University of Hong Kong, Hong Kong, Hong Kong SAR, China

**Keywords:** Bereavement, Depression, Mental health, Nurses, Religion

## Abstract

**Background:**

This paper is to examine the associations between religion, bereavement and depression among nursing professionals using a cross-sectional survey design. There is little empirical evidence in Asia suggesting that religion may either increase or lower the likelihood of nursing professionals being depressed.

**Methods:**

We analyzed the results of a Mental Health Survey soliciting data from 850 Hong Kong nurses (aged 21–59, 178 males) regarding their mental well-being and associated factors, including participants’ socio-economic profile and recent life-events. Multiple linear regression analyses examined associations between religion, bereavement and depression.

**Results:**

Religious faith is weakly associated with lower self-reported depression in bereavement.

**Conclusions:**

Our findings confirm those studies suggesting that religion positively affects mental health and yet healthcare providers have yet to assimilate this insight.

## Background

The population in Hong Kong exceeded 7 million at the end of 2014 [1]. According to the Basic Law of the Hong Kong Special Administrative Region (HKSAR), all Hong Kong citizens can practice any religion. It has been estimated that approximately 43% of the Hong Kong population practice some form of religion [2]. Buddhism and Taoism are the predominant faiths in this metropolitan city [2]. However, most research investigating connections between faith and depression has been conducted in Western countries on cohorts living in the West [3]. There is little empirical evidence in Asia suggesting that religion may either increase or lower the likelihood of people—either employed, unemployed or in any specific profession—being depressed.

### Religion and depression

A growing body of empirical evidence suggests that having a religion is closely associated with better physical and mental health [4], in particular in inhibiting major depression and suicide [5]. Religious congregants show a reduced risk of developing depression [6] and recover quicker [7]. Scholarship has explained these findings through a “buffering hypothesis”: through religious activities, individuals are supported by others within networks [8]. These social ties help reduce social isolation and guard against major mental disorder [4, 6]. Religion also induces optimism and instills hope [4] in providing a coherent framework to interpret life-events [3].

Koenig et al. [3] have recently examined 339 studies (272 cross-sectional, 45 prospective studies, and 22 clinical trials) of the relationship; of the 272 cross-sectional studies, 63% (n = 170) reported a significant positive relationship between religiousness and lower rates of depression; 22% (n = 60) reported no association; 6% (n = 17) reported greater depression among the more religious; and 8% (n = 22) reported mixed findings. 1% (n = 3) reported findings that were complex to interpret. Some researchers look more specifically at the association between religious attendance and depression. Ellision and Flannelly [9] found no relationships between these terms in a national sample of African-Americans. Ellision’s findings were echoed by Smith et al.’s [10] meta-analysis of 147 studies covering some 100,000 subjects; this found a weak negative correlation (−0.096) between religiousness and depressive symptoms after controlling for gender as a confounding variable. Other researchers argued that some religious individuals lacking reassuring conceptual frameworks were more vulnerable to the onset of psychiatric disorder [11]. Other researchers studied the physically and mentally ill population. Koenig [7] followed 1000 depressed serious medically ill inpatients aged 50 years and older either suffering from congestive heart failure or chronic pulmonary disease and depressive disorder. The religious characteristics of these patients were compared with a non-depressed control group (n = 428). Koenig concluded that the severity of depressive symptoms among the religious was inversely related to religious indicators (religious affiliation, prayer, reading scripture). Previous studies have understood religion as a resource helping patients with medical and physical illness cope.

Other researchers concerned themselves with the likelihood of religious people suffering from depression. Some studies show religiosity is associated with lower odds [12–16]. Other research showed increased odds or no association [17]—again highlighting the inconsistency of researchers’ conclusions and failure to arrive at a consensus on the relations between religion and depression.

### Religion and bereavement

How does religion relate to the experience of bereavement? Becker et al.’s [18] systematic review of 32 quantitative and qualitative studies found 94% of studies showed some positive effects of religion on bereavement (22 studies reported positive effects; 6 limited positive effects; 2 no positive effects and one suggested religion aggravated depression). Wortmann and Park [19] recently reviewed 73 articles assessing the relationship between religion and bereavement quantitatively. Having a religion helped people adjust to bereavement. Their later [20] systematic review of qualitative studies found that the experience of loss in bereavement may lead to shifts in fundamental beliefs, including change in and loss of religious faith.

Some researchers focusing on the loss of significant others (next of kin, parents, spouse, children, siblings) have yielded inconsistent results regarding the association of religion and grief. Austin et al.’s [21] study of 57 such bereaved persons found no effect for religiosity as measured by Beck’s Depression Inventory and Shepard Scale but, interestingly, the bereaved subjectively reported fewer depressive symptoms. Recent evidence suggests that having a religion may mitigate stress and depression and facilitate recovery from bereavement [3]. Bereavements cause pain and stress [22]. They may also bring to light contradictions between individuals’ global beliefs (e.g. God protects humans from harm) and goals (e.g. a desire to carry on living with significant others). Such discrepancies can give rise to unhappy feelings or anger or blaming God [23]. At the same time, religion may encourage the bereaved to reflect on religious worldviews—with the effect that if bereavement does not mean the loss of their faith, it may end up deepening it and tending to spiritual growth [24].

### Depression in nurses

By 2020, depression is projected to become the second most common cause of disability [25]. Women are at higher risk of becoming depressed than their male counterparts [26, 27]. A recent study in China revealed that, among 1592 nurses, 61.7% had mild depressive symptoms and 25.1% moderate to severe symptoms [28].

Yoon and Kim [29] revealed similar results that approximately 38% (n = 441) of registered nurses were experiencing depressive symptoms. Younger or single nurses reported higher levels of depression than that of married counterparts (OR = 2.88, 95% CI 1.32–6.27).

Apart from marital status, being forced to act superficially seemed to be the second strongest predictor of depressive symptoms in nurses (OR = 2.46, 95% CI 1.56–3.86); the third strongest was lack of professional reward or recognition (OR = 1.99, 95% CI 1.07–3.70). Recent epidemiological data suggests that the weighted prevalence estimates of moderate to extremely severe depression among 850 Hong Kong nurses came in at 35.8% (AUTHORS, 2015). Female nurses reported depressive symptoms more often than male counterparts. Marital status, chronic illness, job dissatisfaction, collegial disturbance, physical inactivity, sleep disturbance and poor self-reported mental health were significant correlates of depression in nurses.

Recent local findings from authors [30] have implicated that nurses suffer from individual, interpersonal and work-related stressors and they are at high risk of developing psychiatric morbidity, including depression [25, 31–33]. Nonetheless, there have been a paucity of research on ethnically Chinese nurses’ psychic wellbeing [28]. Nurses are persistently subject to increasing high public and professional expectations which pose mental health risks [34]. Existing research shows a marked interest in the association between religion and mental health (or depression) and between religion and bereavement. Research typically studies the general population and the medically or mentally ill. There does not seem to be any study assessing the association between religion, bereavement and depression in a Chinese population. There is as yet no empirical study examining the prevalence of religion among nursing professionals, nor do we have any evidence for the effects of religion on nurses at times of distress. This study represents a first step in examining the association between religion, bereavement and depression in the nursing workforce in Hong Kong. This paper draws on data reported by Hong Kong nurses for their wellbeing and religious belief to shed light on this relationship.

## Methods

### Aims

This study aims to examine the associations between religion, bereavement and depression among nursing professionals in Hong Kong.

### Design

A web-based cross-sectional self-administrative survey.

### Participants

The sampling frame used in this study was extracted from the Association of the Hong Kong Nursing Staff (AHKNS) which comprised over 50% (n = 24,000) of all Hong Kong nurses registered with the Hong Kong Nursing Council [35]. Nurses joined the Association on a voluntary basis.

### Ethical considerations

The study was approved as a social science project by the Human Research Ethics Committee for Non-Clinical Faculties (HRECNCF) and Institutional Review Board of a local university in Hong Kong.

### Data collection

A total of 16,082 (67%) of the Association’s members have registered their emails. The Association sent out a mass email inviting participation in our survey. Written informed consent was obtained from all participants. Anonymity and confidentiality were ensured. A total of 850 purposive samples aged between 21 and 59 years were recruited over a 4-week period from October 2013 to November 2013. The response rate was 5.3%. Socio-demographic and other work-related information was obtained and the questionnaire comprises nine sections and takes approximately 20 min to complete. The sensitivity of some questions meant a number of emergency helplines were listed on the final page.

### Instrument

#### Depression Anxiety and Stress Scale 21

Depression is assessed by the Depression Anxiety Stress Scale 21 (DASS_21_) [35], a validated, reliable self-report psychological instrument using a 4-point Likert scale measuring past-week depression, anxiety, and stress. DASS_21_ is frequently used to measure the severity of depressive, anxiety and stress symptoms in clinical and non-clinical samples [35–37], possessing well-established psychometric properties in reliably measuring depression, anxiety and stress (at a Cronbach’s alpha 0.91, 0.84 and 0.90 respectively). It is also believed capable of differentiating between depression, anxiety and stress [35, 38]. Each item is scored from 0 to 3. The more severe the symptoms in each dimension, the higher the scores in each subscale. Individuals’ sub-score for depression was obtained from the DASS_21_ to measure the severity of symptoms among respondents. Scores from each dimension were summed up and categorized as ‘normal’, ‘mild’, ‘moderate’, ‘severe’ and ‘extremely severe’, according to the DASS manual [35]. The cut-off score for DASS depression is 10. Scores of 0–9 is considered normal. Scores of ≥10 were categorized as mild, moderate, severe, extremely severe depression according to the DASS manual [35]. The Chinese validated translation of the DASS_21_ was used in our study as participants were predominantly ethnically Chinese. One consequence was the exclusion from the study of non-readers of Chinese.

#### Religious belief

Respondents were asked three single-item questions regarding their religious belief:

1. Do you have a religion? (Yes/no)
2. What is your religion? (1: Catholicism; 2: Christianity; 3: Buddhism; 4: Taoism; 5: Ancestor worship)
3. Is religion important to you? (Yes/no)

Since our study was not originally designed to measure the effects of religion on mental health and bereavement, thus, respondents were not asked for their intrinsic or extrinsic religious involvement in this study.

#### Bereavement

The term “bereavement” was taken to mean the death of first degree relatives, meaning one’s spouse, children, parents, next of kin and siblings, good friends/other relatives in the past year.

Respondents were asked two questions about bereavement:

1. Have any of your first degree relatives passed away in the past 12 months? (Yes/no)
2. Do you have other relatives/good friends who have passed away in the past 12 months? (Yes/no)

### Outcome measure

The study took the DASS_21_ depression sub-score as an outcome measure. Adjusted mean differences were taken to determine variations between religious and non-religious participants bereaved over the past 12 months. The mean difference is significant at 0.05 level (2-tailed).

### Data analysis

Multiple linear regression analysis was used to examine the association between religion, bereavement crisis and the DASS_21_ depression sub-score. Both the crude model and the model controlled for socio-economic variables (gender, age, marital status, household income, chronic illness) were reported, as elsewhere on the grounds that these confounding variables were known to our study as risk factors for depression [30]. The interactions between religion and bereavement on the DASS_21_ depression sub-scores were also examined. The statistical analysis was conducted using SPSS version 23.0 (SPSS Inc., Chicago, IL, USA).

## Results

The gender ratio in the Hong Kong nursing population is 7:1 (F:M) [39] while that in our raw data sample is 4:1 (F:M). Our sample is also comparatively younger. To yield representative findings for the Hong Kong population, the study adjusted prevalence estimates by sampling weights reflecting the size of the population as a whole as suggested by the Hong Kong Nursing Council. Specifically, adjustments were made for gender.

### Socio-demographic characteristics

The majority of the respondents were female (87.6%, n = 672), frontline nurses (87.2%, n = 740). The mean age was between 34 and 44 years old (SD ± 2.79). 55% were married, 43% single and 2% divorced, separated or widowed. 70% had obtained a Bachelor degree or above, earned a monthly household income between HKD 40,000 and 59,000 and were general nurses. Respondents had an average 10–20 years of clinical experience (Table 1).

**Table 1:** Socio-demographic characteristics between Christians and Non-christians

	Christian faith			
	No	(%)	Yes	(%)
Gender				
Male	71	67.6	34	32.4
Female	471	63.2	274	36.8
Age				
21–24	53	68.8	24	31.2
25–34	185	67.5	89	32.5
35–44	176	62.2	107	37.8
45–54	112	60.2	74	39.8
55–64	16	55.2	13	44.8
Education level				
Bachelor degree or above	377	63.4	218	36.6
Associate degree	68	63.0	40	37.0
Secondary school (form 4–7)	98	66.7	49	33.3
Marital status				
Single, never married	233	64.5	128	35.5
Married or cohabitance	296	63.4	171	36.6
Divorced, separated, widowed	13	59.1	9	40.9
Monthly household income (HKD)				
20,000–39,000	157	65.7	82	34.3
40,000–59,000	200	64.1	112	35.9
≥60,000	185	62.1	113	37.9
Specialty				
General nursing	365	61.9	225	38.1
Mental health nursing	178	68.5	82	31.5
Position				
Frontline nurses	478	64.5	263	35.5
Nurse managers	65	59.1	45	40.9
Years of employment				
<10	269	66.9	133	33.1
≥11	274	61.0	175	39.0
Job satisfaction				
No	209	68.1	98	31.9
Yes	333	61.4	209	38.6
Chronic illness				
No	419	63.5	241	36.5
Yes	123	64.7	67	35.3
Bereavement (first degree relative)				
No	524	64.5	288	35.5
Yes	19	50.0	19	50.0
Bereavement (other friends or relatives)				
No	399	66.2	204	33.8
Yes	143	58.1	103	41.9
Smoking				
No	536	64.0	302	36.0
Smoked <6 cigarettes a week	5	55.6	4	44.4
Smoked >7 cigarettes a week	2	66.7	1	33.3
Current drinker				
No	413	63.7	235	36.3
Yes (1–2 times/month)	104	64.2	58	35.8
Yes (daily to few times)	25	64.1	14	35.9
Depressive symptoms				
No	343	62.8	203	37.2
Yes^a^	200	65.6	105	34.4

^a^ DASS_depression score ≥10

A total of 70.9% of the respondents worked in a shift rotation pattern. Less than one quarter of our sample (22.3%) suffered from chronic illness. Around 35% reported sleep problems. In terms of life events, 4.5% reported bereavement of first degree relatives in the past 12 months while 28.9% reported bereavement of other friends/relatives in the past 12 months. Current smokers and alcohol drinkers comprised 1.5 and 24% respectively of the sample (Table 1).

### Religious belief

A total of 357 (42%) respondents reported having a religion, 90% of whom said religion was important to them. The most common religion was Christianity (28.8%, n = 244), followed by Catholicism (7.4%, n = 63), Buddhism (5.1%, n = 43), Taoism (0.7%, n = 6), and Ancestor worship (0.5%, n = 4). Some respondents selected more than one religion.

Since most respondents were either Catholics or Christians, these two groups were collapsed and coded into a Christian category. Buddhist, Taoists and ancestor-worshipers and other respondents without religious belief were coded as non-Christian.

Christians had lower mean scores of depression (Mean = 8.07 ± 8.63) than non-Christians (Mean = 8.57 ± 8.93). Independent sample t tests showed that the mean score of depression between individuals with or without a religion were insignificant (*t* = −0.83, df = 848, *p* > 0.05, 95% CI −1.71, −0.70).

### Bereavement

A total of 37 (4.4%) participants had a first-degree relative who died in the past 12 months. Over a quarter of participants (28.4%, n = 241) had other relatives or good friends dying in the past year.

### Multiple regression analysis

Christians had lower adjusted mean scores of depression (Mean = 8.99 SD 1.26, 95% CI 8.91–9.07) than non-Christians (Mean = 12.28, SD 1.26, 95% CI 12.00–12.36) when bereaved. A statistically significant relationship obtains between Christians and non-Christians in degree of depression after bereavement [β (−4.59), SE 1.35, 95% Wald CI (−7.23, −1.94), Wald χ^2^ = 11.57, df = 1, *p* = 0.001]. The goodness of fit (R squared) was 47.8%.

Table 2 reports results by multiple regression analysis after controlling for age, gender, marital status, household income and chronic illness [β (−4.51), SE 1.30, 95% Wald CI (−7.06, −1.95), Wald χ^2^ = 11.97, df = 1, *p* = 0.001]. Results show that respondents being aged between 25 and 44 and divorced/separated/widowed [β (10.36), SE 4.32, 95% CI (1.89, 18.84), Wald χ^2^ = 5.75, df = 1, *p* = 0.002] were significant correlates in distinguishing the level of depression between the religious and non-religious bereaved. The goodness of fit (R squared) was 52.1%.

**Table 2:** Adjusted depression level between Christians and non-Christians in bereavement

Parameter	β	*SD*	95% Wald CI		Wald χ^2^	df	*p* value
			Lower	Upper			
Intercept	2.51	2.76	−2.91	7.92	0.82	1	0.36
Non-Christians	3.03	1.06	0.95	5.12	8.13	1	0.004**
Christians	0^a^	–	–	–	–	–	–
No death	2.24	1.03	0.23	4.25	4.76	1	0.029*
Death	0^a^	–	–	–	–	–	–
Non-Christians*No death	−4.51	1.30	−7.06	−1.95	11.97	1	0.001**
Non-Christians*Death	0^a^	–	–	–	–	–	–
Christians*No death	0^a^	–	–	–	–	–	–
Christians*Death	0^a^	–	–	–	–	–	–
Male	0.44	0.72	−0.98	1.86	0.37	1	0.56
Female	0^a^	–	–	–	–	–	–
Age							
21–24	1.55	1.98	−2.34	5.44	0.61	1	0.44
25–34	3.88	1.72	0.50	7.25	5.06	1	0.02*
35–44	3.78	1.69	0.47	7.09	5.02	1	0.03*
45–54	2.06	1.72	−1.32	5.44	1.43	1	0.23
55–64	0^a^	–	–	–	–	–	–
Marital status							
Single, never married	1.52	2.22	−2.83	5.86	0.47	1	0.50
Married/cohabiting	−1.22	2.19	−5.51	3.08	0.31	1	0.58
Separated/divorced	10.36	4.32	1.89	18.84	5.75	1	0.02*
Widowed	0^a^	–	–	–	–	–	–
Household income (HKD)							
20,000–24,999	0.96	1.45	−1.87	3.79	0.44	1	0.51
25,000–29,000	−1.75	1.34	−4.38	0.88	1.70	1	0.19
30,000–39,000	0.97	0.97	−0.94	2.88	0.99	1	0.32
40,000–59,000	0.81	0.73	−0.61	2.23	1.25	1	0.26
>60,000	0^a^	–	–	–	–	–	–
Chronic illness	3.78	0.74	2.34	5.22	26.33	1	0.26
No chronic illness	0^a^	–	–	–	–	–	–
Scale	70.263^b^	3.44	63.84	77.33	–	–	–

Dependent variable: DASS depression sub-score

Model: (Intercept), Christianity, Death_binary, Christianity*Death_binary, Gender, Age, Marital Status, Income, Chronic illness

Goodness of fit (R^2^) = 52.1%

* *p* < 0.05, ** *p* < 0.01

^a^ Set to zero because this parameter is redundant

^b^ Maximum likelihood estimate

## Discussion

Buddhism and Taoism, interestingly, did not turn out to be a predominant faith. Christianity, however; stood out to be the most predominant faith among our sample. Some of the local public hospitals were subsidized by Christian funding bodies and thus, these local public hospitals have a chapel in situ where nurses can easily access to whenever they need. Easy accessibility and domination of the Christianity faith in healthcare setting may provide a user-friendly platform to encourage Hong Kong nurses to turn into Christianity. There was a dearth of studies examining the proportion of different religious faith specific to Hong Kong healthcare professionals and thus, comparing the religion preference between general population and health professionals was not plausible. Our study showed that the religion preference seemed to vary between general population and nurse professionals in Hong Kong. Future studies may investigate the association between occupation/profession and religious faith across the globe.

Our study found that when respondents had been bereaved, their having a religion significantly lowered their adjusted depression scores in comparison to bereaved non-religious people. Further, respondents aged between 25 and 44 and either divorced or separated were significantly more likely to be depressed. Only very few respondents (4.4%, n = 37) had been bereaved of first-degree relatives in the past 12 months. More had lived through the deaths of more distant friends and relatives. It is possible that younger respondents had less experience dealing with bereavement than older people amongst respondents as a whole. As a result, those in this age segment may become more vulnerable to depressive symptoms, especially if they feel inadequately supported by their social network.

Married people tend to be closely attached to others—sometimes to children and a community, as well as a partner. Some scholars have postulated connections between marriage and integration into religious communities and customs [40]. Divorced or separated individuals may be more weakly attached to others than those in married relationships. These respondents may receive bereavement as a significant loss further disrupting their ability to bond or form meaningful social ties.

Research in social psychiatry and social psychology tends to place little emphasis on the macro-level mediators and moderators of individual-level relationships [41]. As a result, how macrolevel social processes like religion operate on individual-level relationships remains open to question [40]. Clearly, there is a need to elaborate bi-level models (macro–micro level) to fully understand interactions between individual characteristics and religion, bereavement and depression in different cultural contexts. Cross-national analysis will also be needed to examine whether hypotheses concerning religion-depression relationships for some regions can be usefully generalized, for instance from the West to Asian nations where the effect of faith in shaping personal psychologies is poorly understood [40].

In our sample, 90% of the respondents with a religion declared that religion was important to them. It is likely that individuals with a religion have both intrinsic and extrinsic religious involvement. The social support and networks established via religion build strong social ties among co-religionists. These networks, and the consolation of religion itself, can help buffer religious people from depression following on from bereavement [40]. Some religious people believe in an afterlife, meaning the loss of those they love is not permanent for them. They may interpret bereavement as a form of suffering that will eventually be transmuted to salvation [40]. Prayers and religious rituals may also help them cope, so that bereavement is less traumatic for them than for those without faith.

Our study’s findings are consistent findings with high-profile studies [42–44] suggesting certain aspects of religiosity (public religious involvement, intrinsic religious motivation) may be inversely related to depressive symptoms. Of course, religion is always mediated by individuals’ cultural context [45, 46]. Our study also showed that not-bereaved religious people had lower depression scores than not-bereaved non-believers and our study’s findings support an association between depression and religious faith [3].

Researchers have suggested religion protects people from their vulnerabilities in ordinary life-situations, then becomes a dampener of depression in bereavement. Since the current study is limited by its self-reporting cross-sectional design, it cannot speculate on the causality of the link between religion and depression. In one hypothesis, individuals could turn to religion for comfort, support, hope, and meaning in difficult circumstances usually inducing depression. In another, people become depressed after finding religion, which prevents their condition from becoming more severe or requiring psychiatric admission.

## Strengths of our study

Our study has certain unique strengths. First, to the best of our knowledge, it is the first exploratory study to examine the association between religion, bereavement and psychiatric symptoms among Hong Kong nurses. Second, it features a control group of non-religious respondents whose depressive symptom scores were compared with those of religious respondents dealing with bereavement. Confounding variables were controlled for in regression analysis and compared with non-adjusted measurement outcomes. Our findings confirm studies suggesting religion positively affects mental health, specifically in highlighting its role as a moderator of depression for the bereaved.

## Limitations

Despite these strengths, the study is limited. Its cross-sectional design means that data analysis only describes those statistical relationships between religion, bereavement and depression present in our data. We cannot make any causal inferences concerning relationships—which would require a longitudinal design. Respondents’ self-reports of their incidence of depression in the study need to be treated with caution, in that they were not validated by any objective measurement of depression using a standardized clinical diagnostic manual (e.g. the Structured Clinical Interview for Depression). Further, reports were limited to a period of 1 week before returning the survey.

Second, less than half the participants (41.4%) in the study expressed some sort of religious belief. This is lower than in recent surveys conducted both in the USA and in the UK (54 and 70% respectively) [47, 48]. Nevertheless, we must emphasize that our study is not purely a study of religious nurses in dealing with depression and bereavement but rather, we attempted to disentangle the association between religion, bereavement and depression among Hong Kong nurses. The infrequency of believers other than some form of Christians in the study means it lacks the statistical power to examine associations between other religions, bereavement and depression.

Third, the dataset was not originally constructed for the present study. In consequence, intrinsic (prayers, rituals, experiences) and extrinsic religiosity (affiliation, frequency of church attendance, level of social support) were not measured. Hence, participants’ self-report of the importance of religious belief cannot be validated by intrinsic/extrinsic religious involvement. It is difficult to make a causal inference on the impact of religion to those bereaved individuals in our study. The risk of over-or-under estimation of the impact of religious faith on those bereaved individuals may also be possible.

Fourth, participants were asked about the bereavement of (their first degree relatives/friends/close relatives) in the past 12 months. Data emerged may also be subject to recall bias. A multi-methodological approach combining quantitative and qualitative methods is recommended for future studies seeking to replicate results.

## Conclusions

Religious bereaved people reported fewer depressive symptoms than the non-religious. Religion seems to be a moderator in thee terms between faith, bereavement and depression. Religion is a major source of hopefulness in Western traditions, though researchers still disagree on whether it insulates people from the effects of depression. Sociologists’ perspectives towards death draw on the notion that a belief in an afterlife is fundamental to promote human hopefulness. Nevertheless, traditional psychiatric or psychological research on hopefulness and hopelessness has yet to assimilate this insight.
